# Factors influencing tourists’ shared bicycle loyalty in Hangzhou, China

**DOI:** 10.3389/fpsyg.2022.1023308

**Published:** 2022-10-24

**Authors:** Bin Zhou, Qihao Xiong, Ping Li, Ling-en Wang, Hu Yu, Jianying Jin

**Affiliations:** ^1^Marine Economic Research Center, Donghai Academy/Yangtze River Delta Ecological Civilization Research Center/Department of Tourism, Ningbo University, Ningbo, China; ^2^HNU-ASU Joint International Tourism College, Hainan University, Haikou, China; ^3^Institute of Geographic Sciences and Natural Resources, Chinese Academy of Sciences (CAS), Beijing, China; ^4^College of Tourism, Universitat de les Illes Balears (UIB), Mallorca, Spain

**Keywords:** shared bicycle, tourist perceptions, tourist loyalty, Hangzhou, transport

## Abstract

Focusing on Hangzhou, a famous tourist city in China, in this study, four regression models were constructed through four items of tourist loyalty to investigate the influence of tourist perceptions and characteristics on male and female tourist loyalty to shared bicycles. A questionnaire survey and ordered logistic regression model techniques were used. Survey data from 467 tourists indicated that there were significant differences between male and female tourists. For male tourists, their willingness to reuse shared bicycles (Models 1 and 2) was positively correlated with ease of access to cycles, environmental awareness, psychological benefit, and management provision; however, their willingness to recommend shared bicycles (Models 3 and 4) was only affected by environmental awareness, psychological benefit, and management provision. Among female tourists, willingness to reuse shared bicycles (Models 1 and 2) was affected by ease of access to cycles, environmental awareness, and rule adherence, while their willingness to recommend shared bicycles (Models 3 and 4) was affected by ease of access to cycles, environmental awareness, psychological benefit, and managerial provision. In addition, female tourists’ socio-demographic and behavioral characteristics had a significant impact on their loyalty, among which length of stay in Hangzhou and education were significant in the four regression models, and number of visits to Hangzhou had a positive impact on female tourists’ willingness to reuse (Models 1 and 2). In addition, female tourists who used Hellobike had higher willingness to reuse and recommend than those who used Mobike. For male tourists, only length of stay in Hangzhou had a significant impact on their reuse intention (Model 2). The current study extends the theory of attribution to explore the factors which may affect tourist’s loyalty to bicycle-sharing from the perspectives of tourists’ perceptions. It will provide further support to decision makers in the development of new shared-bicycle systems at Chinese tourist destinations, with the aim of strengthening tourist loyalty to shared-bicycle programs.

## Introduction

The sharing economy is a social economy model that distributes a good or service through online platforms in the form of rentals, exchanges, collective purchases, and co-creation (Zhou et al., 2022). As a new type of economic model, the sharing economy has resulted from social technology and economic progress (Wang and Nicolau, 2017), and it is expected to facilitate innovative online activity and generate billions of US dollars globally by 2025 (Keogh et al., 2020). Under the sharing economy model, owners can reap economic benefits through effective allocation of excess resources, thus promoting the sustainable use of resources (Alrawadieh and Alrawadieh, 2018). Bicycle sharing is a new form of transport born out of the sharing economy and has been gradually accepted as one of the main transportation modes aimed at solving the last-mile problem (Guidon et al., 2020).

As a viable alternative to motorized vehicles, bicycle sharing is often associated with the image of a progressive, civilized, and environmentally friendly city (Chen and Huang, 2021). In the field of tourism, the emergence of bicycle sharing has provided tourists with a convenient means of transportation that has contributed to solving their travel problems and is of great value to the development of urban tourism (Bieliński et al., 2019). Studies have shown that bicycle sharing plays an important role in the movement of tourists, especially within 300 meters (about 1,000 ft.) of attractions (Yang et al., 2021). Nevertheless, the shared bicycle utilization rate remains low. According to the monitoring data reported by the Beijing Municipal Traffic Commission (2019), less than 50% of all registered bicycle users are active, and more than half of the bicycles in the shared-bicycle program are unused. Not only is this a serious waste of social resources, but also results in challenges for shared bicycle companies in terms of survival and development (Zhu et al., 2020). Therefore, how to improve the shared-bicycle utilization rate, as well as enhance user loyalty is a key issue to be considered.

However, a research gap still exists in the customer loyalty literature in the bicycle-sharing context. Loyalty is an integral factor in the survival and development of enterprises (Antón et al., 2017). Highly loyal customers will purchase products repeatedly, irrespective of prices, and actively recommend the products to others (Cetin, 2020). Furthermore, as an effective theory for studying customer experience and customer behavior, the attribution theory has been widely used by scholars to study customer loyalty (Kim and Baker, 2020; Nguyen et al., 2021; Osakwe and Yusuf, 2021). This theory argues that customers are rational information processors, and their behavior is formed by making causal inferences consistent with their experience of the product and the information obtained from external sources (Osakwe and Yusuf, 2021). Thus, the factors influencing customer behavior are different due to differences in product attributes as well as externally obtained information. However, there are no similar studies in the emerging field of bicycle-sharing. In addition, due to varied travel purposes, economic levels, consumption patterns, and departure times, different groups of passengers have different travel requirements (Bi et al., 2020). Unlike regular customers, tourists are more experience-oriented, less price-sensitive, and more tolerant of products and services (Song et al., 2019). Many studies have identified significant differences in the process of loyalty formation and its influencing factors between tourists and non-tourists (Chang et al., 2014; Báez-Montenegro and Devesa-Fernández, 2017). However, the relationship between tourists’ perception of the bicycle sharing experience and their loyalty is still unclear. Therefore, this study aims to fill this research gap by using the attribution theory perspective to explore the impact of tourists’ perceptions of attributes of the bicycle sharing experience on their loyalty.

Additionally, researchers have determined that tourists’ demographic statistical variables (Lynn, 1991; Lee et al., 2017; Purani et al., 2019; Rasoolimanesh et al., 2021) and behavior variables (Moliner-Velázquez et al., 2019) significantly impact their loyalty. However, no similar studies in the field of bicycle sharing have been conducted. Therefore, this study uses the attribution theory to investigate the factors that affect tourists’ loyalty to bicycle-sharing from the perspective of tourists’ perceptions. Using a questionnaire and an ordered logistic regression model, we aim to: (1) identify tourists’ perceptions of the bicycle-sharing experience, (2) investigate the influence of tourists’ perceptions on tourists’ loyalty, (3) determine whether tourists’ demographic and behavioral variables affect their loyalty to bicycle-sharing. The remainder of the paper is organized as follows. Section 2 reviews the literatures on bicycle-sharing, tourist loyalty and tourist perception. Section 3 describes the research process, section 4 evaluates the results and the research hypotheses, and section 5 presents conclusions and policy implications.

## Literature review

### Bicycle-sharing

In recent years, bicycle sharing programs have been considered a healthier, convenient, affordable, and environmentally friendly public transport mode in cities across the globe, the use of which will facilitate the improvement of public transportation systems through last mile connectivity (Shaheen et al., 2010; Parkes et al., 2013). People have a wide range of reasons for choosing bicycle sharing for commuting and tourism (Chen and Huang, 2021). Many tourists favor bicycle sharing due to its convenience, as bicycle rental stations are often available within close proximity of tourist attractions (Fishman et al., 2013). Some tourists also enjoy the authentic experience of acting like a local (Chen and Huang, 2021). Moreover, shared-bicycle programs can serve as an effective tool to improve the overall attractiveness of a destination (Davies et al., 2020; Nguyen et al., 2021).

Tourism products and services are considered to be experience-driven, and the tourist experience is one of the most important research components in tourism research (Chen and Huang, 2021). It has been demonstrated that tourists’ experiences significantly impacted tourists’ behavior. For example, Mohamad (2022) concluded that tourists’ experience of the quality of electric train services significantly impacted their loyalty. Zhou et al. (2022) determined that tourists’ experience of the quality and value of online car-hailing had a significant impact on both their satisfaction and loyalty. Regarding bicycle sharing, scholars have also conducted a great deal of research, but most studies focus on tourist demand for and behavior when using bicycle-sharing services (Fishman et al., 2013; Kaplan et al., 2015; Morton, 2018; Nguyen et al., 2021). There is still a lack of empirical research evidence on tourists’ experiences of using the bicycle-sharing services at destinations and its impact on tourist behavior. Understanding the interdependence between tourists’ decisions on activities, attractions visited, and transport modes used can provide a better understanding of tourists’ behavior at destinations (Chen and Huang, 2021). Therefore, this study used the attribution theory to explain how tourists understand their bicycle-sharing experience and how these perceptions affect their loyalty.

### Tourist loyalty and tourist perception

Much of the marketing literature identifies customer loyalty as a deep commitment to repurchasing a product or service, regardless of the attractions other competitors may offer (Oliver, 1999; Oliver, 2014). While recognizing the unique features of shared-bicycle programs, tourist loyalty means that tourists prefer to increase ridership and continue using a public shared-bicycle transport service without seeking or shifting to alternative options and that they are also likely to recommend the service to new users (Webb, 2010). It is worth noting that researchers have been focusing on exploring tourists’ understanding of loyalty, as they have determined that increased tourist loyalty boosts ridership (Lai and Chen, 2011). However, to further attain and sustain tourist loyalty, the factors that influence tourist loyalty when using shared bicycles must be identified and understood (Vicente et al., 2020).

Customer experience is considered one of the determinants of customer behavior and is widely discussed by academics (Uzir et al., 2020; Uzir et al., 2021). According to the attribution theory, the attributes of an object that a person experiences provide grounds for the person to generate perceptions and make appraisals of its overall performance, which result in the person’s follow-up actions (Feldman, 1981). Several scholars have used the attribution theory to explore tourist loyalty (Moon et al., 2021). However, the formation of tourist loyalty is not identical in different contexts (for example, in the fields of hotels, shopping, and transport). For example, Ha et al. (2019) identified five factors associated with customer loyalty in public transport: accessibility, reliability, perceived value, comfort, safety, and security. For some possessions of environmentally friendly products, scholars would argue the role of valuing their green attributes (Chen and Chiu, 2016; Al Halbusi et al., 2020). For instance, Vicente et al. (2020) introduced a new concept of commitment to environmental sustainability when exploring the impact of factors that affect passenger loyalty to public transport services. However, there is a lack of similar research on bicycle-sharing. Therefore, this study uses attribution theory to determine the impact of tourists’ perceptions of the bicycle-sharing experience on their loyalty.

## Methodology

### Study site

Hangzhou, the capital city of Zhejiang Province, is also one of the central cities in the Yangtze River Delta, with strong accessibility to external transportation. As a famous tourist city in China, Hangzhou has many tourists from all over the world. In 2017, Hangzhou received 162.87 million tourists, with total tourism revenue of 304.13 billion yuan (US$45.97 billion in 2017; Hangzhou Bureau of Statistics, 2018). This provides convenient conditions for the study and can ensure the diversity and representativeness of tourists. In addition, Hangzhou has a highly developed bicycle-sharing industry. With the large-scale launch of Ofo, Hellobike, and other major bicycle-sharing companies, the number of shared bicycles in Hangzhou’s six districts increased sharply to over 220,000 vehicles in April 2017. In November 2017, the number of shared bicycles in Hangzhou peaked at 882,700, and the daily order volume was between 800,000 and 1 million (Zhejiang Provincial People's Government, 2017). The high level of bicycle-sharing services attracts many tourists, which facilitates the researchers’ search for target groups.

### Survey design

Based on previous related studies, a questionnaire survey was developed for data collection (Appendix 1). All items were measured on a 7-point Likert-type scale ranging from 1 (strongly disagree) to 7 (strongly agree). The survey consisted of two parts: (1) tourists’ perceptions and tourists’ loyalty to shared bicycles and (2) their socio-demographic information and behavioral characteristics. Tourists’ perceptions of bicycle-sharing reuse were measured by 26 items contextualized for this study (see Appendix 1). Socio-demographic characteristics included gender, age, occupation, education, marital status, monthly income, and number of children. We also asked respondents about their experiences with bicycle sharing, including companions, number of visits to Hangzhou, means of transportation to Hangzhou, length of stay, brand of bicycle used, and frequency of bicycle usage in Hangzhou.

The questionnaire was written in English and translated into Chinese. Using back-translation, English and Chinese language professionals verified that the translation accurately reflected the original text. A pilot survey was administered from October 21 to 22, 2017 before formal distribution. A total of 158 questionnaires were distributed and 152 were recovered (the recovery rate was 96.2%). To ensure validity and reliability, exploratory factor analysis (EFA) was conducted on the usable samples (n = 152) from the pilot test. Items with low factor loadings and cross-loadings were removed after the EFA. Following further expert review, the wording and expression of some questionnaire items were revised. Eventually, six dimensions were extracted: ease of access to bicycles, perceived risk, environmental awareness, psychological benefits, managerial provision, and perceived rule adherence. The cumulative variance contribution rate was 70.13%, which was 60% higher than the minimum acceptable standard.

### Data collection and analysis

A field investigation was conducted to collect questionnaire data for this study. The survey was conducted from October 28 to 31 and November 2 to 5, 2017. Hangzhou West Lake, Lingyin Temple, Xixi National Wetland Park, Southern Song Dynasty Royal Street, and Hefang Street were among the survey sites. These five sites typically have large tourist flows and widespread bicycle-sharing usage and conducting the research in multiple sites made the sample more representative. This study used a simple random procedure targeting tourists with experience using shared bicycles. Normally, every fifth person that passed by was invited to participate in the study to ensure a random sample. We informed the participants that the survey was anonymous and that their personal information would be kept confidential. To exclude Hangzhou residents, potential interviewees’ tourist status and frequency of bicycle sharing were confirmed before the questionnaires were distributed. A total of 600 questionnaires were distributed, and after removing incomplete questionnaires and those that were not taken seriously, 552 valid questionnaires were collected, with an effective rate of 92%.

## Results

### Respondents’ profile

Table 1 shows that the proportion of female respondents (54.2%) is close to that of male respondents (45.8%). Regarding age, most respondents belong to the 19–25 or 26–35 age groups, accounting for 85.2% of the total. Most respondents have a high level of education (57.4% undergraduates and 14.6% postgraduates), which ensure that respondents can understand the question items. Regarding occupation, 30.6% were students, followed by private enterprises (27.6%). Most respondents were traveling with friends (57.8), followed by family (25.5). As for monthly income, 76.7% of the respondents believed that their income was the average of their region. Most respondents came to Hangzhou multiple times (43.8%) and stayed for at least 2 days (60.4%). Most respondents (64.7%) arrived in Hangzhou by high-speed rail, followed by self-driving (20.8%). Concerning the bicycle-sharing service used, most tourists chose Mobike, followed by Ofo and Hellobike, accounting for 46.5, 26.6, and 23.8%, respectively. Among them, 29.1% of the respondents used shared bicycles more than three times during their stay in Hangzhou, followed by 20.1% who used them twice; 50.7% of the respondents used them only once. These data indicate that tourists using bicycle-sharing are mainly young, highly educated, and have an average monthly income. These data are close to the characteristics of the bicycle-sharing user group (ASKCI Consulting Co. Ltd., 2017), which indicates the representativeness of the data.

**Table 1:** The descriptive statistics of sample characteristics (*n* = 467).

Variable	Category	*N*	%	Variable	Category	*N*	%
Gender	Male	214	45.8	Age	12–18	19	4.1
	Female	253	54.2		19–25	186	39.8
Education	Junior high school and below	9	1.9		26–35	212	45.4
	Senior high school	28	6		36–45	39	8.4
	Junior college	94	20.1		46–55	9	1.9
	Undergraduate college	268	57.4		>56	2	0.4
	Postgraduate	68	14.6	Occupation	Civil servant	21	4.5
Marital status	Unmarried	204	43.7		State-owned enterprise	73	15.6
	Married	251	53.7		Private enterprise	129	27.6
	Separated/divorced	12	2.6		Public institution	65	13.9
Number of children	0	245	52.5		Student	143	30.6
	1	168	36		Free lance	23	4.9
	2	52	11.1		Retired	4	0.9
	3 or more	2	0.4		Other	9	1.9
Monthly income level	Far below average	24	5.1	Companion	Alone	40	8.6
	Below average	46	9.9		Family	119	25.5
	Average	358	76.7		Friend	270	57.8
	Above average	30	6.4		Tour group	28	6.0
	Far above average	9	1.9		Other	8	1.7
Length of stays	1 day but not overnight	136	29.1	Means of transportation to Hangzhou	Self – driving	97	20.8
	2	217	46.5		High-speed railway	302	64.7
	3	65	13.9		Bus	54	11.6
	4 or more	46	9.9		Other	11	2.4
Types of cycles used	Mobike	217	46.5	Number of visits to Hangzhou	1	106	22.7
	Ofo	124	26.6		2	148	31.7
	HelloBike	111	23.8		3 or more	213	45.6
	Xiaoming bicycle	32	6.9	Frequency of cycles used in Hangzhou.	1	237	50.7
	Zhixiang bicycle	1	0.2		2	94	20.1
	Xiaobai bicycle	3	0.6		3	66	14.1
					4 or more	70	15

**Figure 1:**
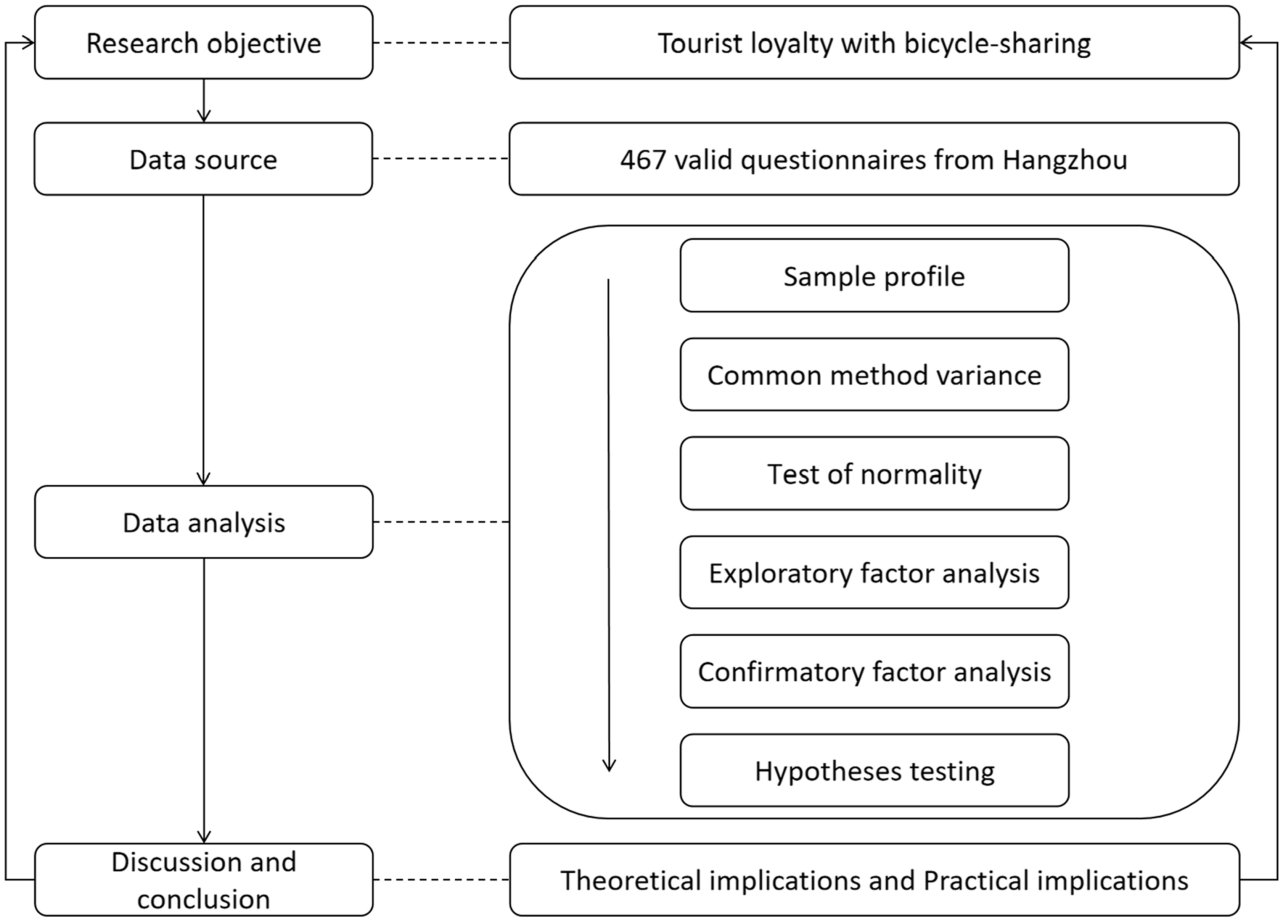
Road map of the research.

The road map of the research is shown in Figure 1.

### Common method variance

This study first used the Harman’s single factor test to examine the common method variance (CMV) of the data. After fixing the number of factors to 1, the overall variance explained by the first common factor was 37.884%, which was lower than the empirical criterion of 40%. Therefore, we believe that CMV was not a serious problem in this study.

### Measurement model test

**Figure 2:**
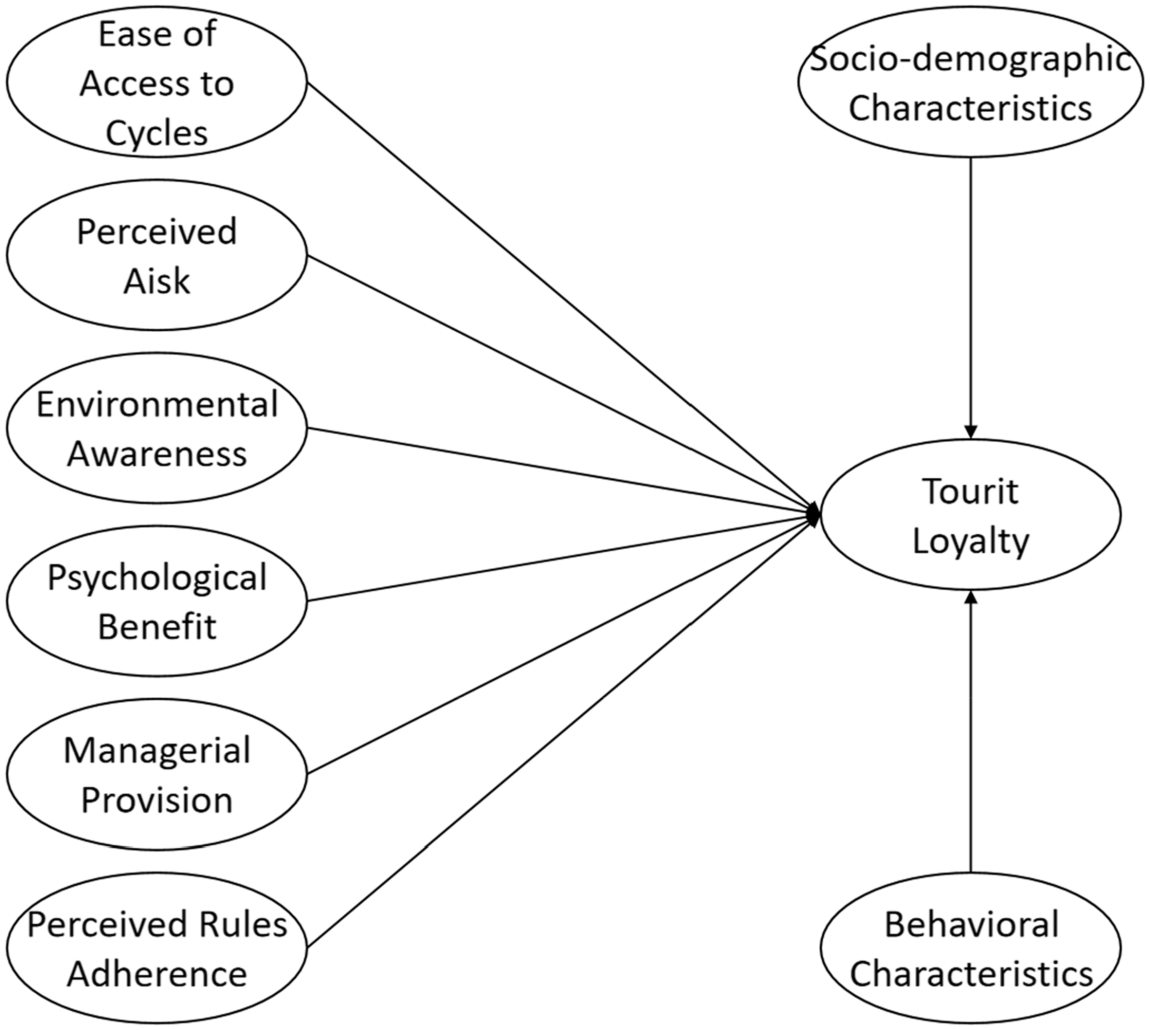
Theoretical model.

First, it was necessary to check the normal distribution of the data. The results indicate that the skewness values of all items range from −1.272 to −0.247, and the absolute value is <3. The kurtosis of all items ranges from −0.497 to 2.664, and the absolute value is <8. According to Kim (2013), the data in this study conform to the normal distribution and can thus be analyzed (Figure 2).

The original dataset was randomly split into two subsets for cross-validation of the measurement. The software SPSS 25.0 and Amos 22.0 were used for EFA and confirmatory factor analysis (CFA). EFA was performed on one subsample (n = 233), while CFA was performed on the other (n = 234).

Through SPSS 25.0, EFA was conducted by using the principal component method with VARIMAX rotation. The factor number was decided with the rule of the extracted eigenvalue being higher than 1. A six-factor underlying structure was identified which explained 70.348% of the total variance (Table 2). The factor loadings for all items exceeded 0.5. The Cronbach’s alpha of the total scale was 0.921, and the KMO value was 0.877 (> 0.7), which passed Bartlett’s sphericity test (df = 325; *p* < 0.001). The alphas for the six factors were 0.840, 0.909, 0.920, 0.815, 0.828, and 0.763, all above 0.70. These proved that the scale had good reliability (Hair et al., 2010). After considering factor loadings and item descriptions, the six factors were named ease of access to cycles, perceived risk, environmental awareness, psychological benefit, managerial provision, and perceived rule adherence.

**Table 2:** The results of EFA (*n* = 233).

Items	Latent variables						Mean	S.D.	Min	Max	Variance explained	Cronbach’s alpha
	1	2	3	4	5	6						
A1			0.754				6.00	0.573	4	7	12.736%	0.840
A2			0.756				5.94	0.623	4	7		
A3			0.736				6.02	0.672	4	7		
A4			0.704				5.98	0.651	4	7		
A5			0.649				6.00	0.648	4	7		
A6			0.536				5.82	0.760	3	7		
A7	0.853						5.92	0.821	3	7	14.794%	0.909
A8	0.856						5.94	0.825	3	7		
A9	0.840						5.94	0.822	3	7		
A10	0.824						5.94	0.800	3	7		
A11	0.698						5.98	0.839	3	7		
A12		0.906					6.46	0.688	4	7	12.978%	0.920
A13		0.882					6.38	0.746	4	7		
A14		0.860					6.45	0.693	4	7		
A15		0.763					6.40	0.749	4	7		
A16				0.827			6.01	0.832	3	7	11.361	0.815
A17				0.793			6.08	0.875	3	7		
A18				0.695			6.21	0.867	3	7		
A19				0.521			5.64	0.963	1	7		
A20					0.835		5.77	0.935	3	7	10.210%	0.828
A21					0.774		5.73	0.925	3	7		
A22					0.659		5.78	0.860	2	7		
A23					0.591		5.80	0.909	3	7		
A24						0.812	6.07	0.782	3	7	8.269%	0.763
A25						0.750	6.09	0.749	3	7		
A26						0.560	6.26	0.609	4	7		

KMO = 0.877, *χ*2 = 3934.088, *df* = 325, *p* < 0.000, Total variance explained = 70.348%, Total Cronbach’s α = 0.921.

CFA was performed by Amos 22.0 to further validate the measurement structure of tourists’ perceptions of bicycle sharing, based on the EFA results. As presented in Table 3, the CR value for each of the six constructs ranged from 0.791 to 0.945. This indicated the internal consistency reliability of the measures.

**Table 3:** The results of CFA (*n* = 234).

Common fators	Items	Factor loading	Mean	S.D.	Min	Max	AVE	CR
Factor 1 Ease of access to cycles	X1	0.722	5.98	0.690	3	7	0.563	0.885
	X2	0.805	5.87	0.706	3	7		
	X3	0.712	5.97	0.758	3	7		
	X4	0.766	5.94	0.724	3	7		
	X5	0.822	5.96	0.692	3	7		
	X6	0.663	5.83	0.798	3	7		
Factor 2 Perceived risk	X7	0.934	5.78	1.028	2	7	0.688	0.916
	X8	0.912	5.89	0.923	3	7		
	X9	0.839	5.87	0.969	3	7		
	X10	0.778	5.82	0.906	3	7		
	X11	0.654	5.91	0.980	2	7		
Factor 3 Environmental awareness	X12	0.964	6.35	0.773	3	7	0.813	0.945
	X13	0.883	6.31	0.855	3	7		
	X14	0.936	6.32	0.788	3	7		
	X15	0.817	6.27	0.844	3	7		
Factor 4 Psychological benefit	X16	0.854	5.92	0.892	3	7	0.590	0.849
	X17	0.882	5.93	0.846	3	7		
	X18	0.731	6.06	0.882	4	7		
	X19	0.564	5.39	1.052	3	7		
Factor 5 Managerial provision	X20	0.862	5.63	1.021	3	7	0.611	0.860
	X21	0.901	5.65	0.966	3	7		
	X22	0.710	5.73	0.885	2	7		
	X23	0.622	5.71	1.052	2	7		
Factor 6 Perceived rules adherence	X24	0.833	6.05	0.782	3	7	0.560	0.791
	X25	0.727	6.07	0.738	3	7		
	X26	0.676	6.26	0.703	3	7		

RMR = 0.047, SRMR = 0.061, GFI = 0.840, NFI = 0.871, IFI = 0.927, CFI = 0.927, RMSEA = 0.069.

The factor loading of items was between 0.564 and 0.964. The AVE value of all factors was greater than 0.5. All fitness indexes were acceptable (RMR = 0.047, SRMR = 0.061, GFI = 0.840, NFI = 0.871, IFI = 0.927, CFI = 0.927, RMSEA = 0.069), with SRMR lower than 0.08, GFI, and NFI greater than 0.8. In addition, χ2/df was 2.120, which was less than the standard value of 3. These results confirmed the presence of convergent validity between these six constructs (Hair et al., 2010).

To evaluate discriminant validity the cross-loadings of the indicators were examined and the square root of the AVE values was compared with the construct’s correlations. The results presented in Table 4 indicate sufficient discriminant validity between the six constructs (Fornell and Larcker, 1981).

**Table 4:** The results of discriminant validity (*n* =  234).

	AVE	Ease of access to cycles	Perceived risk	Environmental awareness	Psychological benefit	Managerial provision	Perceived rules adherence
Ease of access to cycles	0.563	**0.750**					
Perceived risk	0.688	0.424	**0.829**				
Environmental awareness	0.813	0.404	0.388	**0.902**			
Psychological benefit	0.590	0.519	0.456	0.299	**0.768**		
Managerial provision	0.611	0.508	0.471	0.403	0.63	**0.782**	
Perceived rules adherence	0.560	0.445	0.387	0.543	0.331	0.483	**0.748**

Bold value is the arithmetic square root of AVE.

### Research hypotheses

Compared with cars, bicycles are more convenient because they offer accessible, multimodal, flexible, and spontaneous transport solutions (Zademach and Musch, 2018). Tourists are free to rent and return bicycles for a reasonably short time in the destination (Bieliński et al., 2019). When tourists can easily find a shared bicycle that they can use, they will have a great experience; conversely they may feel frustrated (Fishman et al., 2015). Hence, an appealing bicycle rental or a good parking experience is one of the main attraction for tourists (Yuan et al., 2019). Based on the above discussion, we propose the following hypothesis:

*H1:* Ease of access to bicycles exerts a significant positive impact on tourist loyalty to bicycle-sharing services.

The shared-bicycle system provides tourists with a useful way to move around at a destination easily and flexibly, while exploring an authentic experience by acting like a local (Chen and Huang, 2021). Additionally, cycling is a physical activity that can both improve tourists’ physical and mental health (Xu et al., 2019). Tourists can relax, exercise, and experience reduced feelings of pressure while riding (Zhou et al., 2020). These experiences can play a positive role in the formation of tourist loyalty (Chen and Huang, 2021). Therefore, we propose the following hypothesis:

*H2:* Psychological benefits exert a significant positive impact on tourists' loyalty to bicycle-sharing services.

For many reasons, including the government’s response to pollution, an increasing number of tourists want to choose an environmentally friendly means of transportation. Tourists’ environmental awareness has a positive impact on their behavior (Yusof et al., 2016). For example, Han et al. (2009) determined that tourists with environmentally friendly attitudes tend to show loyalty to environmentally friendly products. They are more supportive of a company when they identify with its social responsibilities (Niu et al., 2016). Therefore, we propose the following hypothesis:

*H3:* Environmental awareness exerts a significant positive impact on tourist loyalty to bicycle-sharing services.

Risk is inherent in every decision but significantly impacts the tourism industry (Joo et al., 2021). For example, bicycles-sharing tourists face non-refundable deposits, potential vehicle damage, and traffic safety risks. Perceived risk refers to the spirit cost associated with customers’ purchasing behavior, which significantly impacts tourist behavior (Nguyen et al., 2021). Tourists’ perceived risk is highly correlated with their experience and loyalty (Zhang and Tang, 2021). When their perceived risk is low, they are more at ease with bicycle-sharing and more inclined to become loyal, whereas, when they have a higher risk perception, they tend to be less loyal and refuse to reuse (Jin et al., 2016). Thus, the following hypothesis is proposed:

*H4:* Perceived risk exerts a significant negative impact on tourist loyalty to bicycle-sharing services.

Research has demonstrated that tourists’ compliance with traffic and city regulations during bicycle-sharing significantly affects their experience (Zhou et al., 2020). Xie (2018) came to a similar conclusion that customers’ self-restrictions, such as compliance with traffic rules and bicycle-use norms, can effectively enhance their experience. According to the self-control theory, a person with high self-control derives higher satisfaction from moral rectitude and cultural value achievement (Hofmann et al., 2014). Tourists with high self-control use bicycle sharing more civilly, receive higher satisfaction levels, and are more inclined to be loyal to bicycle sharing. Therefore, we propose the following hypothesis:

*H5:* Perceived rule adherence exerts a significant positive impact on tourist loyalty to bicycle-sharing services.

Scholars from different fields have verified that tourists’ perceptions of enterprise management quality and service quality have a significant impact on loyalty (Han et al., 2021). Tourists’ trust in a company’s management ability directly affects their willingness to utilize the services provided by the company (Li et al., 2022). Tourists’ trust in and experience of services are key factors influencing their post-consumption behavior (Moise et al., 2020). Therefore, we propose the following hypothesis:

*H6:* Managerial provision exerts a significant positive impact on tourist loyalty to bicycle-sharing services.

The formation of loyalty is not as simple as commonly believed (Kumar et al., 2013). For example, Rasoolimanesh et al. (2021) discovered that female tourists have more complex, sufficient configurations and heterogeneity than males, which leads to significant differences in loyalty formation. In addition, different age groups have different values, characteristics, and behaviors, and different levels of acceptance of new things, which significantly impact their loyalty (Martinović and Barišić, 2018; Purani et al., 2019). It has also been determined that educational level has a significant impact on tourist loyalty (Lee et al., 2017). Various researchers also believe that in addition to tourists’ socio-demographic characteristics, behavioral characteristics—such as trip purpose, visit frequency, and online reviews—also significantly impact tourist loyalty (Moliner-Velázquez et al., 2019). Therefore, we propose the following hypothesis:

*H7:* Tourists' socio-demographic and behavioral characteristics exert a significant positive impact on tourist loyalty to bicycle-sharing services.

### Conceptual model test

To further explore the causal relationship between each tourist perception factor and tourist loyalty to bicycle-sharing, we constructed the following regression model (Formula 1) by Stata 16.0. Because the dependent variable tourist loyalty (Y) consisted of ordered and discrete data measured on a Likert scale, this study employed ordered logistic regression analysis. We established four regression models based on the four items of tourist loyalty. In addition, we divided the respondents into male and female groups and studied the differences and similarities of influencing factors of loyalty between them. The model established linear functions of explanatory variables (*x_i_* and *z_j_*) related to the dependent variable tourist loyalty Y*_n_* (*n* = 1,2,3,4) where *x_i_* (*i* = 1,2,3,4,5,6) represents tourists’ perceptions of shared-bicycle rental, comprising six variables: Ease of access to bicycles, perceived risk, environmental awareness, psychological benefits, managerial provision, and perceived rule adherence (*z_j_* represented tourists’ socio-demographic and behavioral characteristics).


(1) $$Y_{n}=\alpha_{0}+\beta_{i}x_{i}+\gamma_{i}z_{i}+\varepsilon_{i}$$

According to the above analysis, the independent variables include six dimensions of tourist perception and 13 socio-demographic variables. The results indicated that the coefficients of many independent variables were not significant, requiring the gradual deletion of those independent variables with insignificant effects. Finally, four significant independent variables—education, length of stay in Hangzhou, means of transportation to Hangzhou, and brand of bicycle used—were retained.

As shown in Table 5, the pseudo R2 coefficients of the models were between 0.217 and 0.281, and the significance sig. Values were all 0.000. The fit information passed the Chi-square test, indicating a significant correlation between variables; the degree of fit of each model was acceptable.

**Table 5:** Ordered logistic regression analysis model results (*n* = 467).

Variables	Model 1		Model 2		Model 3		Model 4	
	Female	Male	Female	Male	Female	Male	Female	Male
Ease of access to cycles	1.041 (0.327)	1.028 (0.354)	0.951 (0.317)	0.981 (0.358)	0.741 (0.322)	NS	0.728 (0.328)	NS
Perceived risk	0.433 (0.219)	NS	NS	NS	NS	NS	NS	NS
Environmental awareness	0.581 (0.249)	1.009 (0.27)	0.659 (0.252)	0.672 (0.257)	0.640 (0.253)	1.077 (0.272)	NS	1.127 (0.273)
Psychological benefit	NS	0.591 (0.262)	NS	0.728 (0.263)	0.685 (0.287)	0.898 (0.27)	0.951 (0.298)	0.920 (0.269)
Managerial provision	NS	0.536 (0.261)	NS	NS	0.868 (0.284)	0.806 (0.263)	0.741 (0.285)	0.705 (0.261)
Perceived rules adherence	0.574 (0.287)	NS	0.688 (0.280)	NS	NS	NS	NS	NS
Number of visits to Hangzhou	−0.379 (0.193)	NS	−0.508 (0.191)	NS	NS	NS	NS	NS
Length of stay in Hangzhou	0.494 (0.166)	NS	0.381 (0.162)	0.509 (0.187)	0.614 (0.172)	NS	0.500 (0.169)	NS
Education	0.329 (0.160)	NS	0.317 (0.152)	NS	0.533 (0.165)	NS	0.494 (0.166)	NS
Hellobike *VS* Mobike	−0.669 (0.334)	NS	−0.807 (0.327)	NS	−0.804 (0.335)	NS	−0.706 (0.337)	NS
Chi-square	127.280	117.010	109.750	96.150	149.160	108.040	147.480	112.250
Pseudo R2	0.244	0.260	0.206	0.217	0.278	0.246	0.281	0.249
Log likelihood	−197.058	−166.838	−211.159	−173.593	−193.472	−165.465	−189.171	−169.622

Standard errors in parentheses. NS: not significant at the 5% level.

### Gender-based differences in tourist loyalty between male and female groups

As shown in Table 5, four items were used in this study to measure tourist loyalty, among which item 1 (Model 1) and item 2 (Model 2) measured tourists’ willingness to reuse shared bicycles and item 3 (Model 3) and item 4 (Model 4) measured tourists’ willingness to recommend shared bicycles.

According to Models 1 and 2, ease of access to cycles had a significant positive impact on both male and female tourists’ willingness to reuse shared bicycles. According to Models 3 and 4, ease of access to cycles only had a significant positive impact on female tourists’ willingness to recommend shared bicycles. Therefore, H1 was partially supported. Perceived risk was not significant to both male and female tourists’ willingness to reuse and recommend, thus, H2 was not supported (one result contrary to the hypothesis). Environmental awareness had a significant positive impact on both male and female tourists’ willingness to reuse and recommend, thus, H3 was supported. According to Models 1 and 2, psychological benefit and managerial provision had no significant impact on female tourists’ willingness to reuse, but had a significant positive impact on that of male tourists. In addition, psychological benefit and managerial provision had a significantly positive impact on the recommendation intentions of both male and female tourists. Thus, H4 and H5 were partially supported. Perceived rule adherence had a significant positive impact solely on female tourists’ willingness to reuse. Therefore, H6 was partially supported.

### Effect of socio-demographic and behavioral characteristics

For male tourists, only length of stay in Hangzhou had a significant positive impact on their willingness to reuse. For female tourists, length of stay in Hangzhou and education had a significant positive impact on their willingness to reuse and recommend. Number of visits to Hangzhou had a significant negative impact on female tourists’ willingness to reuse. In addition, compared with female tourists who used Mobike, those who used Hellobike had higher willingness to reuse and recommend.

## Discussion and conclusion

This study fills some theoretical gaps and enriches the literature on tourists’ perception and loyalty of shared bicycle services. Although scholars have discussed customer experience and behavior extensively (Chang et al., 2014; Báez-Montenegro and Devesa-Fernández, 2017; Song et al., 2019; Al Halbusi et al., 2022), this is the first time that the relationship between customer perceptions of their bicycle-sharing experience and loyalty has been discussed in depth from the attribution theory perspective. This extends the theoretical research on attribution theory and provides empirical evidence for future research on bicycle-sharing. In addition, while previous studies have discussed customer loyalty to bicycle sharing (Zhou and Zhang, 2019; Liu et al., 2020), they seem to assume that consumers are homogeneous and ignore their heterogeneity. This study adopted the perspective of tourists and systematically explored the impact of tourists’ perceptions of bicycle-sharing and their socio-demographic and behavioral characteristics on their loyalty to bicycle sharing.

The results of this study indicate that tourists’ perceptions regarding ease of access to bicycles, environmental awareness, psychological benefits, and managerial provision all have significant positive effects on their loyalty to shared bicycle services. This confirms the findings of previous studies that tourist perception has a significant positive impact on tourist loyalty (Chang et al., 2014; Báez-Montenegro and Devesa-Fernández, 2017; Song et al., 2019). In addition, in this study, we grouped tourists according to gender and included demographic and behavioral variables in the regression models. The results of the study indicate that tourists’ gender, age, number of visits to Hangzhou, length of stay in Hangzhou, and type of bicycle used all had a significant direct impact on tourist loyalty. These results are significant as they provide insights into improving the tourist transport experience, enhancing the destination image, and promoting the development of sharing economies.

Ease of access to cycles has a significant positive impact on tourist loyalty to shared bicycles. This corresponds with the findings of Park et al. (2021) that accessibility is an important influencing factor on passenger loyalty to public transport. As one of the most famous cities in China, Hangzhou is an important tourist attraction, boasting of the most advanced bicycle-sharing system worldwide (Li et al., 2017). The flexibility of the bicycle sharing system effectively solves the problem of tourists having to change their mode of transportation, providing Hangzhou tourists with a very competitive choice in terms of time and cost (Saberi et al., 2018).

The stronger tourists’ environmental awareness, the stronger their loyalty to bicycle sharing. The knowledge that bicycle sharing could reduce the use of private cars—effectively curbing environmental pollution and the urban traffic problems caused by self-driving—brings tourists psychological satisfaction and a sense of accomplishment in terms of engagement with green consumption, with the added benefit of physical exercise (Zhu et al., 2020). This is consistent with the results reported by Chen and Chiu (2016) and Chen and Huang (2021), which claimed that tourists’ awareness of the tourism environment creates a sense of achievement regarding their reuse of shared bicycles. The more environmentally conscious they are, the higher their loyalty to “consume green.”

Bicycle sharing can evoke positive emotions, such as happiness, in tourists (Zhou et al., 2020). It also represents a new consumer fashion, a status that will increase tourists’ willingness to reuse, which is consistent with the results of Godovykh and Tasci (2021). Strong psychological benefits have a significant positive impact on tourists’ willingness to reuse. Additionally, tourists who use bicycles when traveling can slow their pace, linger in scenic spots, better interact with locals and other tourists, and explore tourist destinations in depth, all of which can provide tourists with a better travel experience and increase their loyalty to bicycle sharing.

Managerial provision has the most significant positive impact on tourist loyalty. As a shared mode of transport, the reasonable price of shared bicycles (RMB 1.50 per 30 min, 1 RMB = 0.1436 USD) attracts tourists to use them, while the high service quality encourages tourists’ loyalty to them through the mediating variable of product satisfaction (Zhou and Zhang, 2019; Shen and Yahya, 2021). Tourist safety is a paramount consideration in the daily maintenance of bicycle sharing, its sustainability, and management. Therefore, these factors affect tourists’ trust in shared-bicycle safety and, in turn, their willingness to reuse this means of transportation.

Among tourists’ socio-demographic characteristics, gender, educational background, and behavioral characteristics (such as number of visit to Hangzhou, length of stay in Hangzhou, mode of transportation to Hangzhou, and the brand of bicycle used) significantly impact their willingness to reuse the bicycle-sharing. These findings are consistent with those of previous studies (Lee et al., 2017; Martinović and Barišić, 2018; Moliner-Velázquez et al., 2019; Purani et al., 2019; Rasoolimanesh et al., 2021); however, this study determined that some variability still exists in the area of bicycle sharing. Firstly, there are significant differences between male and female tourists regarding the factors influencing their willingness to reuse and recommend. Female tourists focus more on the safety attributes of bicycle-sharing, while male tourists focus more on its health attributes. Secondly, male tourists’ loyalty appeared unaffected by their socio-demographic and behavioral characteristics. As for female tourists, high education levels, extended stay, and the low number of visits are closely linked to their high level of loyalty. Finally, this study found that Mobike was more popular among the tourists surveyed and that tourists who used Mobike were more inclined to become loyal. This may be because Mobike began operations earlier and has a relatively higher convergence rate in Hangzhou and a better brand image.

### Practical implications

According to their path coefficients, managerial provision and ease of access to cycles mostly impact tourists’ loyalty to bicycle-sharing services. Therefore, enterprises need to improve the quality of leasing services to protect their rights and interests. For example, the quality of shared bicycles and maintenance and dispatching services should be strengthened to improve the overall performance of the shared-bicycle program. In addition, a standardized management system, such as detailed parking regulations and usage norms, can also ensure that tourists can find bicycles more easily and increase their loyalty. Therefore, managers can regulate bicycle-sharing parking spots, and improve the connection between other public transportation systems and bicycle-sharing.

Psychological benefits and environmental awareness have a significant positive impact on tourists’ loyalty. Managers should promote more green attributes and health attributes of shared bicycles. For example, they can cooperate with the government to plan some exclusive routes for bicycle riding near scenic spots to create an environment that advocates civilized cycling and green travel across society as a whole. This will not only improve the image of the city but also enhance tourists’ loyalty to bicycle-sharing. In addition, tourists should be guided to act courteously when bicycle sharing and enhance their self-management ability. High perceived rules adherence is also closely related to high loyalty.

This study also found that tourists’ socio-demographic and behavioral characteristics significantly impact their loyalty. Managers can develop different marketing strategies for different tourist groups. For example, managers can promote the safety benefits of bicycle-sharing to female tourists while promoting the physical benefits of bicycle-sharing to male tourists. Managers must also focus on highly educated tourist groups, who tend to be more loyal. Managers can offer them special events, discounts, memberships, and so on. Tourists who revisit more often and those who travel by high-speed rail are more likely to be loyal to bicycle sharing. Companies can, therefore, work with map navigation apps to target tourists based on their behavior and target specific tourists with coupons to entice them to use shared bicycles. The study also found that the brand of shared bicycles significantly affected tourists’ willingness to reuse. Therefore, shared-bicycle companies need to learn from one another’s management and marketing methods to make their products more attractive to tourists and increase the reuse rate.

### Research limitations and future work

This study examines tourists’ perceptions related to shared bicycles using the literature method. In future studies, this method could be accompanied with interviewing, network text analysis, the questionnaire experiment method, and other approaches to further expand the source of perception items. Additionally, the network questionnaire survey method may be used to enrich the source of questionnaire data. In future studies, mixed research methods and fsQCA could also be used to analyze the loyalty of film and television tourists. A follow-up study should be conducted to compare tourists’ willingness to use shared bicycles before and after their most recent use. A significant limitation of this empirical study is that it was conducted in Hangzhou, a famous tourist city in China, which has a developed economy. The nature of this location affects, to some extent, the universality of the research results. Therefore, famous tourist cities such as Guilin, Dali, and Lijiang (located in the economically underdeveloped areas of China) should be used for comparative research. The perception factors that affect tourists’ use of shared bicycles should be re-selected to suit the context of these locations. Moreover, this study only selected interviewees from among domestic tourists in Hangzhou. In future studies, foreign tourists and local residents could also be recruited as participants to conduct a comparative study.

## Data availability statement

The raw data supporting the conclusions of this article will be made available by the authors, without undue reservation.

## Author contributions

BZ and L-eW: methodology, software and validation, formal analysis, and original draft preparation. BZ, QX, and PL: writing—review and editing. HY and JJ: visualization. BZ: supervision. L-eW: funding acquisition. All authors contributed to the article and approved the submitted version.

## Funding

This study is supported by the National Natural Science Foundation of China (grant no. 42171223), Youth Innovation Promotion Association, Chinese Academy of Sciences to L-eW.

## Conflict of interest

The authors declare that the research was conducted in the absence of any commercial or financial relationships that could be construed as a potential conflict of interest.

## Publisher’s note

All claims expressed in this article are solely those of the authors and do not necessarily represent those of their affiliated organizations, or those of the publisher, the editors and the reviewers. Any product that may be evaluated in this article, or claim that may be made by its manufacturer, is not guaranteed or endorsed by the publisher.

## Appendix 1

**Table:**

Latent variables	Items	Source
**Tourists’ perception**		
Factor 1 Ease of access to cycles	X1 I think it’s easy to find a bicycle sharing.	Eboli and Mazzulla (2010)
	X2 I think it is convenient to get a bicycle sharing.	Pattarakitham (2015)
	X3 I think the layout of bicycle sharing is reasonable.	Eboli and Mazzulla (2010)
	X4 I think bicycle sharing can save time.	Pattarakitham (2015), Guo et al. (2017)
	X5 I think bicycle sharing is unobstructed.	Titze et al. (2008), Eboli and Mazzulla (2010)
	X6 I think the number of bicycle sharing is sufficient.	Eboli and Mazzulla (2010)
Factor 2 Perceived risk	X7 I think there are potential traffic safety hazards in bicycle sharing.	Zhang et al. (2017)
	X8 I think the registration identity information has the risk of being exposed.	Pavlou et al. (2007), Zhang et al. (2017)
	X9 I think there is a security risk in using bicycle sharing deposit.	Forsythe et al. (2006), Zhang et al. (2017)
	X10 I think there is a risk of damage in using bicycle sharing.	Dholakia (2001), Zhang et al. (2017)
	X11 I think there is a risk of damage in using bicycle sharing.	
Factor 3 Environmental wareness	X12 I think bicycle sharing is in line with the concept of low carbon.	Fishman et al. (2013)
	X13 I think bicycle sharing can improve awareness of environmental protection.	
	X14 I think bicycle sharing reduces environmental pollution.	
	X15 I think bicycle sharing is in line with the concept of green consumption.	
Factor 4 Psychological benefit	X16 I am happy to share bicycle sharing with others.	Guo et al. (2017)
	X17 I am interested in using bicycle sharing.	De Vries and Carlson (2014), Kaplan et al. (2015), Zhang et al. (2017)
	X18 Using bicycle sharing makes me happy.	De Vries and Carlson (2014), Zhang et al. (2017)
	X19 I feel very fashionable in using bicycles sharing.	
Factor 5 Managerial provision	X20 I think bicycle sharing has been orderly placed.	Shaheen et al. (2014)
	X21 I think the bicycle sharing charge is reasonable.	
	X22 I recognize the service quality of bicycle sharing operators.	
	X23 I recognize the daily maintenance of bicycle sharing operators.	
Factor 6 Perceived rules adherence	X24 I think I used a shared bicycle in a civilized way	Bailey et al. (2016)
	X25 I think I obey city management regulations when using bicycles sharing.	Titze et al. (2008)
	X26 I think I obey traffic regulations when using bicycles sharing.	
Tourist loyalty		
	L1 I would like to use bicycles sharing again	Hwang et al. (2021), Nguyen-Phuoc et al. (2021)
	L2 I am highly probable to use bicycles sharing again.	
	L3 I would recommend bicycles sharing to friends and family.	
	L4 I would encourage friends and family to use bicycles sharing.	
